# A Forced Damped Oscillation Framework for Undulatory Swimming Provides New Insights into How Propulsion Arises in Active and Passive Swimming

**DOI:** 10.1371/journal.pcbi.1003097

**Published:** 2013-06-13

**Authors:** Amneet Pal Singh Bhalla, Boyce E. Griffith, Neelesh A. Patankar

**Affiliations:** 1 Department of Mechanical Engineering, R. R. McCormick School of Engineering and Applied Science, Northwestern University, Evanston, Illinois, United States of America; 2 Division of Cardiology, New York University School of Medicine, New York City, New York, United States of America; Georgia Institute of Technology, United States of America

## Abstract

A fundamental issue in locomotion is to understand how muscle forcing produces apparently complex deformation kinematics leading to movement of animals like undulatory swimmers. The question of whether complicated muscle forcing is required to create the observed deformation kinematics is central to the understanding of how animals control movement. In this work, a forced damped oscillation framework is applied to a chain-link model for undulatory swimming to understand how forcing leads to deformation and movement. A unified understanding of swimming, caused by muscle contractions (“active” swimming) or by forces imparted by the surrounding fluid (“passive” swimming), is obtained. We show that the forcing triggers the first few deformation modes of the body, which in turn cause the translational motion. We show that relatively simple forcing patterns can trigger seemingly complex deformation kinematics that lead to movement. For given muscle activation, the forcing frequency relative to the natural frequency of the damped oscillator is important for the emergent deformation characteristics of the body. The proposed approach also leads to a qualitative understanding of optimal deformation kinematics for fast swimming. These results, based on a chain-link model of swimming, are confirmed by fully resolved computational fluid dynamics (CFD) simulations. Prior results from the literature on the optimal value of stiffness for maximum speed are explained.

## Author Summary

The damped harmonic oscillator framework has been applied to interrogate numerous engineering systems like the tuned mass damper used in power transmission, automobiles, and buildings to reduce vibrations. We apply the same framework to undulatory swimming to understand the emergence of movement due to muscular and/or environmental forcing. It helps elucidate why flexible bodies can indeed be propelled forward by not only the internal muscular forcing but also by external fluid forces as reported earlier in which dead trout were found to swim in the wake of cylinder. We show how forcing triggers the first few deformation modes of the swimmer similar to how the appropriate forcing triggers the fundamental deformation modes on a guitar string. We show how the lower deformation modes produce the best forward propulsion of the body. This insight reveals that swimming is viable for small enough frequencies of neuromuscular activation relative to the natural frequencies of the body and for sufficiently stiff elastic properties. Thus, these results identify the key mechanistic parameters that would have been crucial to the evolutionary emergence of swimming animals. The proposed framework can help understand neural control of movement in swimming as well as control of underwater vehicles.

## Introduction

The movement of animals like undulatory swimmers is an emergent behavior that starts from muscle activation [1]. There are two primary issues in undulatory swimming. The first issue is to understand how muscle activation leads to the observed deformation kinematics. The second issue is to understand how deformation kinematics produce movement of the body as a whole, e.g., forward translational motion.

Muscle activation, body mass, elastic properties, and interactions with the environment cause the observed deformation of undulatory swimmers. The deformation kinematics of undulatory swimmers can be complex in general. The question of whether precise and complicated activation of muscles is essential to generate specific deformation kinematics is important to understand how these swimmers control their movement. The deformation of the body has been regarded to have two components. The part that is caused by active muscle forcing is the “active” component, whereas the part that results due to fluid forcing is the “passive” component [2], [3]. The active and passive components are strongly coupled [2], [3]. For example, Liao et al. [4] carried out experiments on a dead trout in the wake of a cylinder and observed its swimming motion. Such swimming caused solely by surrounding fluid forces is referred to as “passive swimming”. They found that the “passive swimming” gait was similar to the “active swimming” gait of the trout. Here, swimming caused by muscle activation is referred to as “active swimming.” It is challenging, in general, to separate the similar looking active and passive components of the deformation. According to the active-passive decomposition framework, passive swimming has only passive component of deformation. However, active swimming has both active and passive deformation components because muscle activation as well as external fluid forces are present. A detailed mathematical description of passive motion induced by external vorticies can be found in Alben [5]. Work by Tanabe and Kaneko [6] on a falling paper shows that fluttering and rotation of the falling paper can be periodic or chaotic depending upon the external forcing from the fluid. Active swimming can be due to muscle forcing in a swimming animal or due to active motor torques in a robotic swimmer [7]–[12]. The observed body deformations are a response to the combined internal (muscle/motor) and external (fluid) forcing which dictates the swimming behavior of a system.

The question of how deformation kinematics lead to forward translational motion is crucial to understand optimal conditions for movement. Some swimming gaits are optimal for fast swimming (during prey capture or predator escape), some are optimal for conserving energy (keeping a fixed station), whereas some optimal gaits combine both these objectives (traveling long distances). Several studies on optimal swimming motion in the noninertial regime have been done in the past [13]–[17]. For example, Spagnollie and Lauga [18] showed that the helical shape for an infinitely long flagellum leads to the fastest swimming motion. Pironneau and Katz [19] studied the optimal flagellar undulations that lead to minimal energy expenditure. Avron et al. [20] used conformal mapping techniques to find optimal two-dimensional microswimmers. Optimized artifical microswimmers have been studied by Dreyfus et al [21]. Wilkening and Hosoi [22] analyzed optimal shapes of a swimming sheet at low Reynolds number using analytical and numerical techniques. A comprehensive review of swimming at low Reynolds number and optimal conditions in the noninertial regime can be found in Lauga and Powers [23]. In comparison, an understanding of optimal conditions for swimming in the inertial regime is much less developed.

There are some key differences in swimming in the noninertial and inertial regimes. For example, in the noninertial regime, the power required to generate body deformations at each instant is fully dissipated into the surrounding fluid. However, in the inertial regime some part of the power is required to accelerate the body. Analysis of optimal swimming motion in the inertial regime is further complicated by nonlinear effects. Kern and Koumoutsakos [24] used fully resolved CFD techniques based upon unstructured grids to obtain the optimized kinematics of a two- and three-dimensional eel for fast and efficient swimming. Tytell et al. [1] used an immersed body technique to understand the effect of body stiffness and inertia on the motion of an undulatory swimmer. McMillen et al. [25] used a calcium dynamics model for muscle contractions to understand the swimming motion of an anguilliform swimmer in the inertial regime. In spite of this progress, complicated interactions between muscle forcing, hydrodynamic forces, inertia, and elastic properties of a swimmer make it difficult to identify the key control parameters that lead to optimal swimming.

In this work a chain-link model [25]–[28] of undulatory swimming is used as a model system to interrogate the emergence of deformation and movement in response to forcing. We consider swimming to be a forced damped oscillation problem. Deformations due to muscles and due to interaction with the environment (fluid) are not differentiated. The system is forced by muscles (active swimming), the surrounding fluid (passive swimming), or both, among various possibilities. It is shown that a body has fundamental modes of deformation with corresponding natural frequencies. Forcing triggers the first few deformation modes of the body. An understanding emerges of how the deformation in turn generates forward translational motion of the body. Relatively simple forcing patterns can trigger complex deformation kinematics that lead to movement. For a given muscle activation, the forcing frequency relative to the natural frequency of the damped oscillator is important for the emergent deformation characteristics of the body. We quantitatively show how the power generated from muscle work is used in creating body deformations and then it is dissipated into the surrounding fluid. A closed form expression for swimming velocity is derived by a leading-order analysis of the equations of motion. Optimal kinematics for fast swimming motion are obtained from the closed form expression. This helps identify the key parameters controlling the optimal conditions for fast swimming. The kinematics obtained for fast swimming, even faster than those identified in [24], are verified by performing fully resolved CFD simulations in the nonlinear regime. This approach also leads to an understanding of optimal body stiffness and forcing frequencies of muscle activation.

In the following sections, the forced damped oscillation formulation is presented first. It is then used to elucidate how swimming motion is generated. Examples of active and passive swimming that arise because of different types of forcing of the system are presented including a discussion on why typical swimming motions involves lower deformation modes. The pathway of power transfer during the swimming cycle is discussed thereafter. Optimal deformation kinematics for fast swimming are discussed next, followed by optimal parameters for fast swimming.

## Methods

We present the nonlinear equations of motion, based on drag models, for a generic undulatory swimmer based upon chain-link configuration. We refer to this as a reduced-order model. Leading-order equations of motion are derived thereafter, which unifies the dynamics of an undulatory swimmer in various scenarios (internal muscle activation or external force). A comparison between the leading-order and nonlinear equations of motion is presented for completeness.

### 2.1 A reduced-order model for undulatory swimming

**Figure 1:**
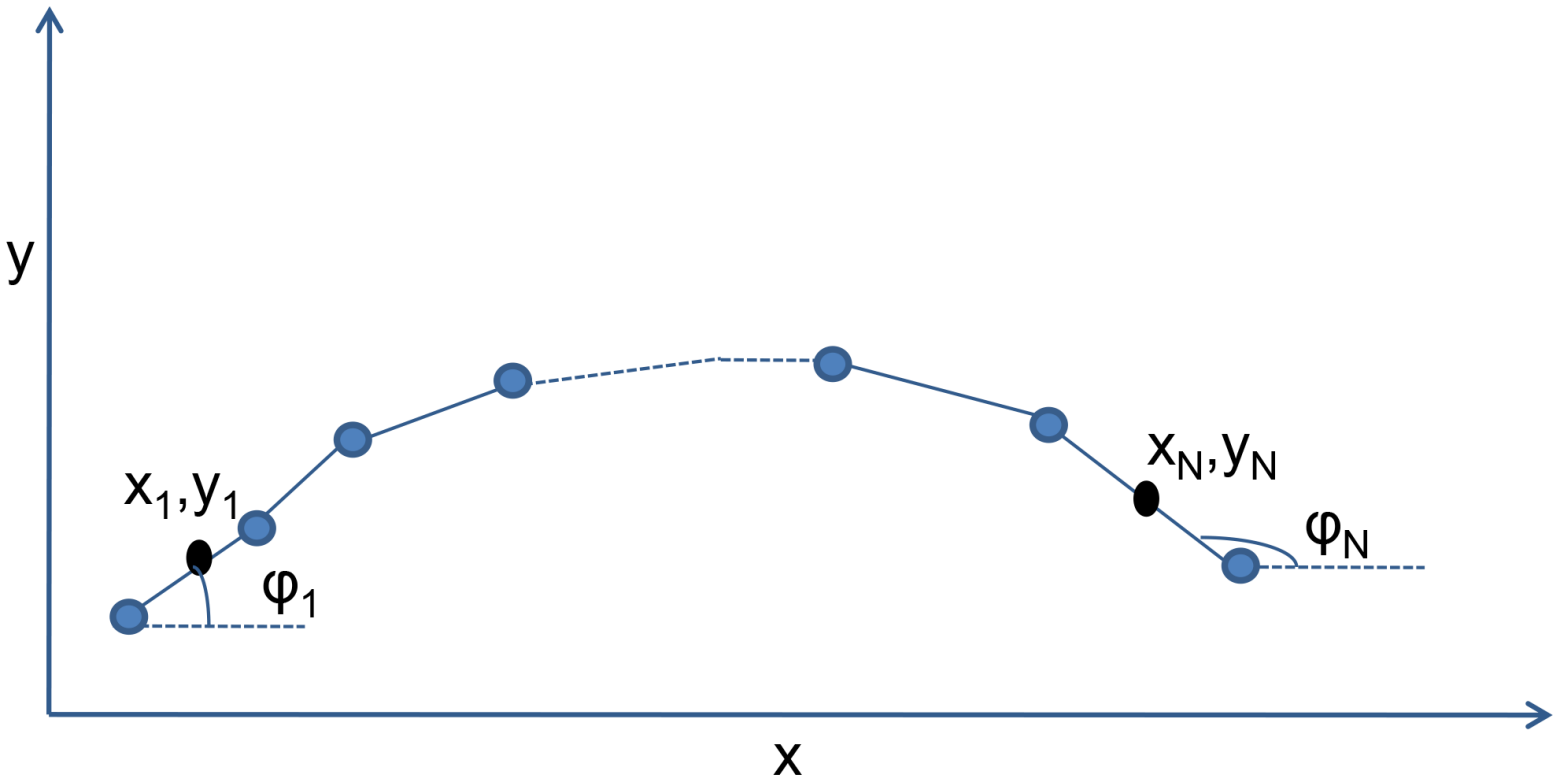
A chain-link model for an undulatory swimmer. Each rigid link is connected to its neighboring link(s) at the common joint by stiff springs in 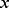 and 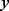 directions. The center of mass of each link and its inclination with the 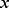 axis is denoted by 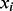, 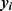, and 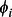, respectively.

We model the body of an undulatory swimmer as a planar chain of interconnected rigid links (Fig. 1) and use a resistive drag model to account for the hydrodynamic forces acting on the body. The rigid links forming the body follow constraint dynamics, i.e., the endpoints of the two adjacent links are forced to lie on top of each other at the common joint. To solve the equations of motion, the forces at the link joints, needed to maintain this constraint, can be eliminated from the equations, and the remaining equations can be solved for the independent variables (velocities of each link). This approach is followed by McMillen et al. in [25], [28]. In our present analysis, we implement this constraint by connecting the links by stiff springs at the joints. We will refer to this approach as the *penalty method* (PM). The stiff springs penalize the system if two links at a common joint try to separate from each other. This approach has been used in the fully resolved immersed body (IB) method of simulations [1], [29]–[32]. Implementing the constraint via stiff springs will help us obtain the leading-order equations of motion (section 2.4). When we are using the resistive drag model (section 2.2) with nonlinear equations of motion, we refer to the model as the nonlinear resistive chain-link PM model. When we are using the resistive drag model with leading-order equations of motion, we will refer to it as the leading-order resistive chain-link PM model.

To derive the nonlinear equations of motion, we isolate a single link which is assumed to be cylindrical in shape of length 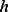 and radius 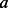. By analyzing the forces and moments on the link, we can write Newton's law of motion for it. The end links are treated differently from the internal links because they have free boundary conditions. For the 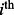 internal link, the equations of motion are written as
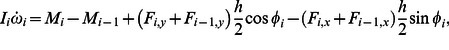
(1)

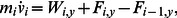
(2)

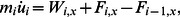
(3)in which 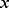 and 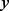 are the axial and transverse directions, respectively. 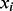, 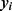 denote the coordinates of the center of mass (COM), 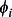 is the angle with the horizontal axis, 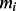 is the mass, and 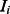 is the rotational inertia of the 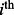 link. 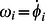 is the angular velocity, 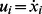 is the axial velocity, and 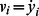 is the transverse velocity of the 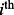 link. Taylor's resistive model [33] is used for the hydrodynamic forces 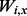 and 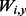 in the axial and transverse directions, respectively. 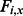 and 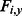 are the forces at the joint in the axial and the transverse directions, respectively, and are written as
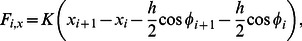
(4)

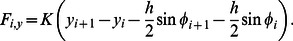
(5)The spring stiffness coefficient 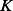 is assumed to be the same in both axial and transverse directions. 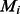 in Eq. (1) is the moment produced by the muscles. Following the model for muscle moment in [28], the moment 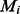 is written as
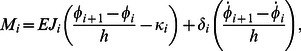
(6)in which 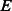 denotes the Young's modulus of the material of the body, 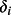 is the viscoelastic damping coefficient, 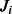 is the cross sectional moment of area of the link, and 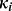 is the preferred curvature at the 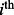 joint. The first term on the right-hand side of Eq. (6) is a model for the activation by the muscles. It is proportional to the difference between the actual curvature with respect to the preferred curvature 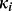. The preferred curvature can be modeled in terms of neuronal models for muscle activation [25]. Those details are not considered in this work. Instead, the preferred curvature is computed based on some given preferred shape of the body. Thus, according to the model, muscle torques are activated when the swimming body tries to match its preferred shape.

### 2.2 Resistive drag models

The drag force depends upon the local velocity of the body relative to the fluid. Depending upon the Reynolds number (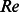), there are different drag models. Taylor [33] used experimental data to propose a drag model for smooth cylinders kept in a perpendicular flow for a range of Reynolds number (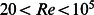). McMillen et al. [28] decomposed the drag force into normal and tangential components for smooth oblique cylinders based upon Taylor's fitting of drag coefficients [33]. According to [28], drag force (per unit length) acting at the COM of smooth oblique cylinder of radius 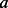 is decomposed into normal and tangential components in terms of the normal and tangential velocities, 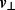 and 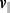, respectively, and is written as
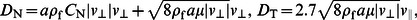
(7)in which 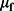 is the viscosity of the fluid, 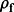 is the fluid density, 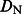 and 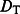 are the drag forces (per unit length) in the normal and tangential directions, respectively. The value of 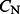, used in Eq. (7) varies between 0.9 to 1.1 for the Reynolds number range 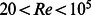. The normal and tangential drag forces can be written as
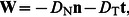
(8)in which **n** and **t** denote the normal and tangential vectors, respectively, to the link.

For low 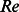 flows, using slender body theory, Lighthill [34] was able to decompose the drag forces into tangential and normal components. He derived expressions for drag coefficients in the normal (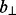) and tangential (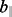) directions as
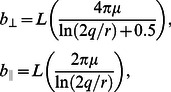
(9)in which 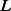 is the body length, 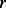 is the body radius and 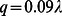 (with 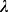 being the wavelength of the undulation). Using this drag model the drag forces on a link at low 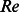 can be written as
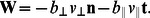
(10)


We use resistive drag models as given by Eq. (8) for intermediate to high Reynolds number (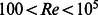) and Eq. (10) for low Reynolds number (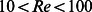). Tytell et al. [1] showed that the lateral forces calculated by the resistive drag model lag behind the lateral forces when calculated by fully resolved CFD simulations. McMillen [25], [28] showed that the resistive drag model is a good approximation to qualitatively capture the forward swimming velocity profile. For the purposes of this work, we use the resistive drag model. This is because while the resistive lateral forces might lag behind the actual lateral forces as shown by Tytell et al. [1], it is the force balance in the axial direction that determines the axial swimming velocity. Even if the reactive force model is used for the thrust component of the axial motion, it should be equated to the resistive drag force in the axial direction according to the theory by Lighthill [35]. In addition, in the average sense, both the reactive and the resistive force models have similar nonlinear characteristics. Finally, as will be shown in this paper, the results obtained from the resistive model are qualitatively consistent with our fully resolved CFD calculations.

### 2.3 Validation of the reduced-order model

Validation of the nonlinear resistive chain-link PM model is done using the parameters reported in [28]. The swimming motion is actuated by a traveling wave of preferred curvature. The preferred shape of the body is assumed to be a backward traveling wave of the form
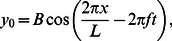
(11)in which 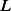 is the bodylength of the fish, 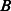 is the amplitude of the traveling wave, and 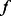 is the frequency of the traveling wave, which is taken to be 2 Hz. The preferred curvature is computed from this preferred shape. The body is assumed to have a uniform circular cross section of radius 1 cm and length 20 cm. This gives a wavespeed 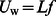 of 40 cm/s. The Young's modulus of the body is taken to be 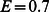 MPa from [28]. The spring stiffness coefficient is taken to be 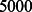 (N/m). This value proved sufficient to keep the two links together at the common joint. The water viscosity and density are taken to be 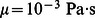 and 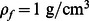. The values of the various parameters are summarized in table 1. For these values, the Reynolds number defined by 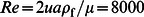. For drag forces, we use Taylor's drag model from Eq. (8), with suitable drag coefficients as in Eq. (7). The body is discretized into 40 links of equal length. To solve the equations of motion (1)–(3) we use MATLAB's built-in time adaptive ODE solver ode45 with time steps bounded to 1 ms.

**Table 1:** Parameter values.

Parameter	Value
Length (L)	20 cm
Links (N)	40
Frequency (f)	2 Hz
Radius (r)	1 cm
Young's modulus (E)	0.7 MPa
Viscosity of water 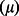	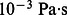
Density of water 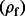	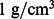

**Figure 2:**
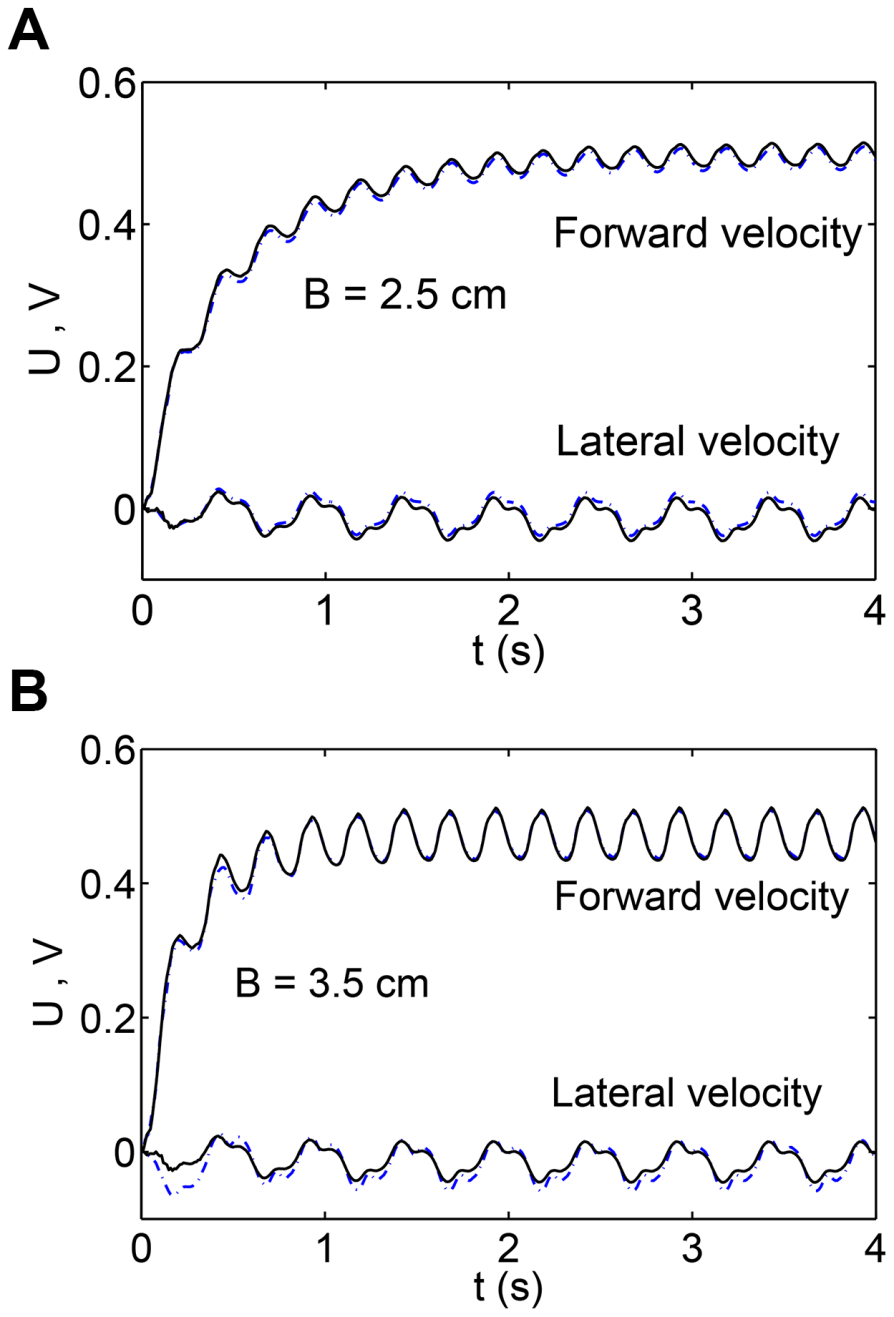
Validation of the nonlinear resistive chain-link PM model. Forward (upper curves) and lateral (lower curves) velocities of the COM of a uniform circular rod following its preferred shape 
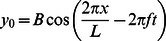
, normalized by the wavespeed (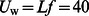 cm/s) are shown in this figure. fig_B25 shows the case when the amplitude B = 2.5 cm and (b) is for the case of B = 3.5 cm. Solid lines denote the PM model and dashed lines denote the model as described in [28]. Taylor's nonlinear resistive drag model is used in these simulations.

To confirm that PM approach gives accurate results, we compared its solution with the approach in [28], in which they used constraints at the joints between the links instead of using stiff springs. Fig. 2 shows the comparison of the PM model with the model in [28]. Two amplitudes of the backward traveling wave are considered (Fig. 2(a) and 2(b)). As can be seen in the figure, the two models are in good agreement with each other.

### 2.4 Leading-order model for undulatory swimming

To gain insight into how muscle activation or external forcing leads to forward translation motion of an undulatory swimmer, leading-order equations of motion are derived. The derivation is based on the assumption of small body deformations. Thus, angles 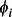 made by the rigid links with the horizontal axis are assumed to be small. We approximate terms like 
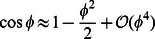
 and 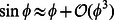.

The leading-order axial momentum equation is derived from Eqs. (3) and (4) as
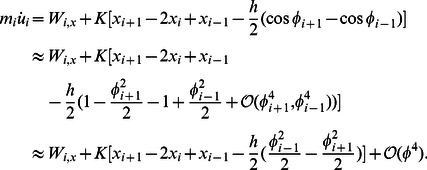
(12)The leading-order transverse momentum equation is derived from Eqs. (2) and (5) as
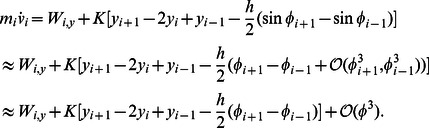
(13)The leading-order rotational momentum equation is derived from Eqs. (1), (4), and (5) as
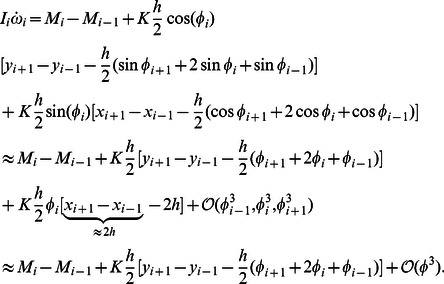
(14)The leading-order analysis of the drag terms, 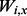 and 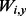, in Eqs. (12) and (13) is performed to obtain the complete set of leading-order equations. Substituiting 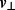 and 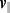 in terms of 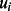 and 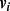, **n** and **t** in terms of 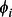 in Eq. (10), we obtain the 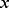 component of the drag force as
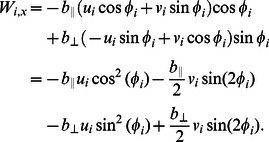
(15)Defining 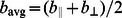, 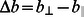 and substituting 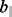 and 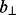 in terms of 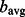 and 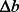 in Eq. (15) we get
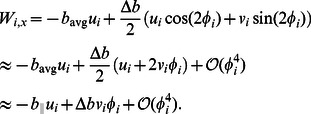
(16)Similarly we can write the leading-order equation for 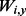 as
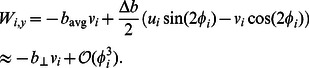
(17)
Eqs. (12), (13) and (14) together with Eqs. (16) and (17) gives us the complete set of leading-order equations of motion. These are summarized below:
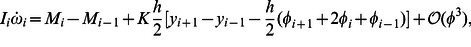
(18)

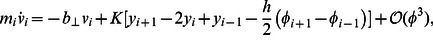
(19)

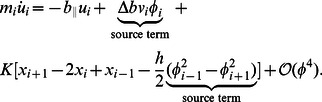
(20)The muscle moment term 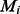 in Eq. (18) is linear in 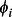. We remark that a nonlinear muscle model with dominant leading-order terms with respect to 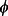 will also fit within this leading-order framework. From Eqs. (18)–(20), it can be seen that to the leading order, the rotational and transverse momentum equations, Eq. (18) and Eq. (19), are coupled to each other, but are independent of the axial velocity equation. However, the axial velocity equation is dependent on the tranverse and rotational velocities due to the “source term” identified in Eq. (20). The leading-order equations show that the axial velocity is lower order (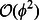) compared to the transverse and rotational velocities (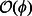). Eqs. (18)–(20) constitute the leading-order forced damped oscillation equations for undulatory swimming.

To derive an expression for the steady swimming velocity of an undulatory swimmer, we consider the leading-order axial momentum equation for the 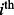 link
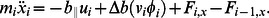
(21)Averaging the above equation over 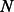 links, leads to the cancellation of the internal spring forces, and gives the equation for the axial velocity of the COM of the body,
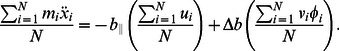
(22)Taking a time-average of the above equation over a single period of the swimming cycle gives
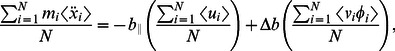
(23)in which 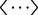 indicates time-average of a quantity. The left-hand side of Eq. (23) vanishes at steady state, and this gives the steady state swimming velocity of an undulatory swimmer as
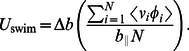
(24)


Eq. (24) indicates that there must be anistropy in drag for the body to swim. This is in agreement with the previous studies on swimming at low Reynolds number [36]. It also shows that the swimming velocity depends upon the time-average of the product of the lateral velocity and the angular position of the body. This term primarily depends on the deformation kinematics. If a nonzero time-averaged lateral velocity and angular position can be provided to the body, then it is able to propel itself forward. The body can swim even if there is no active muscle forcing. For example, if the surrounding fluid can provide the body with appropriate lateral and angular velocities, then it can lead to swimming. This has been observed in the case of a “dead” trout swimming in the wake of a cylinder [4], [37]. Thus, the leading-order equations indicate that the dynamics of swimming are a response to the forcing that goes into the system irrespective of whether the forcing is internal (muscle) or external (surrounding fluid). As will be shown later, the swimming system has fundamental modes of deformation, just like a forced damped oscillator. This is referred to as a unified forced damped oscillation framework for undulatory swimming in this work.

### 2.5 Comparison between the leading-order and nonlinear equations of motion

**Figure 3:**
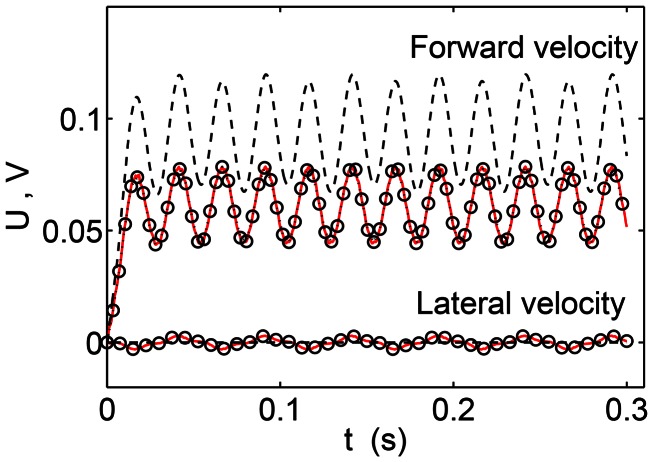
A comparison of three models. The figure shows forward and lateral swimming speeds, 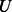 and 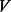, respectively, normalized by the wavespeed 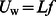. The solid line represents the nonlinear model of [28], circles represent the nonlinear resistive chain-link PM model, and the dashed line represents the leading-order resistive chain-link PM model. The length of the body is taken to be 1 cm and it has a constant cross sectional radius of 0.05 cm. The frequency of the wave passing through the body is taken to be 20 Hz which gives a wavespeed, 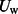, of 20 cm/s. Leading-order drag forces were used in all the three models.

To compare the leading-order system of equations with the nonlinear equations of motion, we take a case of anguilliform swimming at low Reynolds number. The physical parameters are summarized in table 2 and they represent the case of a juvenile zebrafish [38]. The initial configuration of the body is taken to be its intrinsic rest state. Fig. 3 compares the three models. The nonlinear resistive chain-link PM model (circles) agrees well with the model described in [28] (solid line). The nonlinear resistive chain-link PM model solved Eqs. (1)–(3) by using the leading-order drag forces. The nonlinear model of [34] also solved Eqs. (1)–(3) with the leading-order drag forces, but the constraint forces were eliminated instead of modeling them as spring forces. Eqs. (18)–(20) were solved for the leading-order resistive chain-link PM model with leading-order drag forces. As shown by dashed lines in Fig. 3, it gives a higher forward swimming velocity compared to the nonlinear models. The steady state velocity for the leading-order resistive chain-link PM model from the simulation and normalized by the wavespeed is 0.092 (Fig. 3, dashed lines). This is also verified from Eq. (24), in which the computed solution for 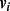 and 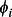 from the simulation is used to compute the normalized steady state swimming velocity. For the nonlinear models, the normalized steady state forward swimming velocity is 0.064 (Fig. 3, dashed and circles). The Reynolds number based upon the swimming speed of juvenile zebrafish is around 20.

**Table 2:** Parameter values.

Parameter	Value
Length (L)	1 cm
Links (N)	40
Frequency (f)	20 Hz
Radius (r)	0.05 cm
Young's modulus (E)	0.7 MPa
Viscosity of water 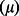	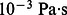
Density of water 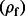	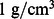

## Results

### 3.1 Passive swimming

The term passive swimming is used here to imply swimming generated by some external forcing, but with no active muscle forcing. The forced damped oscillation formulation, discussed above, shows that as long as there is forcing of the system that leads to appropriate lateral and angular movement (i.e., 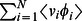 is nonzero), there is swimming. Any type of swimming would fall within this unified framework. To ensure that this is the case we show, in this section, that passive swimming is indeed resolved in this unified framework. Some fundamental scenarios are discussed below.

#### 3.1.1 How can a rigid body swim?

**Figure 4:**
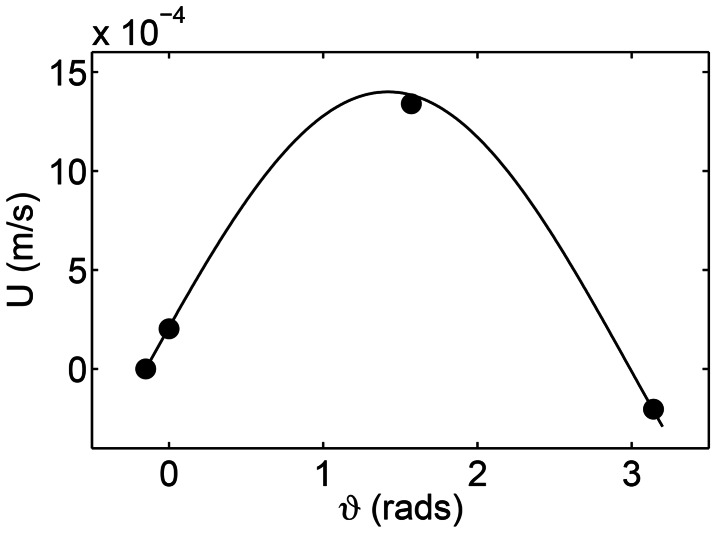
The swimming velocity of a single rigid link in the presence of external force and torque at various phase differences. —: analytical; 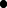: numerical.

Consider a rigid link subjected to an external vertical force 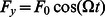 and an external torque 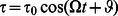, in which 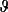 represents the phase difference between the applied force and the torque. Consequently, the link will move up and down and simultaneously undergo a rotational rocking motion. The equations of motion for the link can be written as
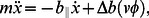
(25)

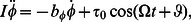
(26)

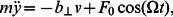
(27)in which 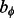 is the rotational damping that acts on the link in Eq. (26). The steady state response of rotational momentum equation (26) is found analytically as
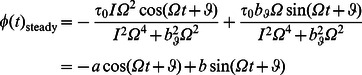
(28)

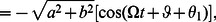
(29)in which 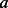 and 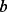 are positive quantities in Eq. (28) and 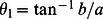 in Eq. (29). The steady state response of transverse momentum equation (27) can be written as
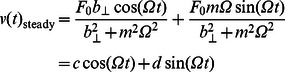
(30)

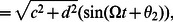
(31)in which 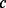 and 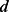 are positive numbers in Eq. (30) and 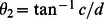 in Eq. (31). At steady swimming the time-average of left-hand side of Eq. (25) vanishes and we obtain the steady swimming velocity of the link as
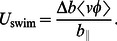
(32)To evaluate the right-hand side of Eq. (32), we multiply Eqs. (28) and (30) and take a time-average of the product to obtain
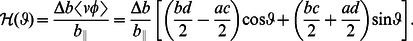
(33)The above equation can be evaluated for various phase differences between the applied vertical force and torque. It can be verified that for the phase difference 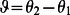, 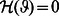. This gives a net zero swimming velocity. To verify the equations derived for the swimming of a single rigid link, we take a rigid link of length 20 cm and radius 1 cm. We apply an external force in the transverse direction, which has an amplitude of 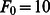 mN, and an external torque of an amplitude 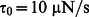 on the link and observe its swimming motion for different phase differences 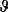. As can be seen in the Fig. 4, depending upon the phase difference between the external torque and transverse force, the link can reach different steady swimming velocities.

**Figure 5:**
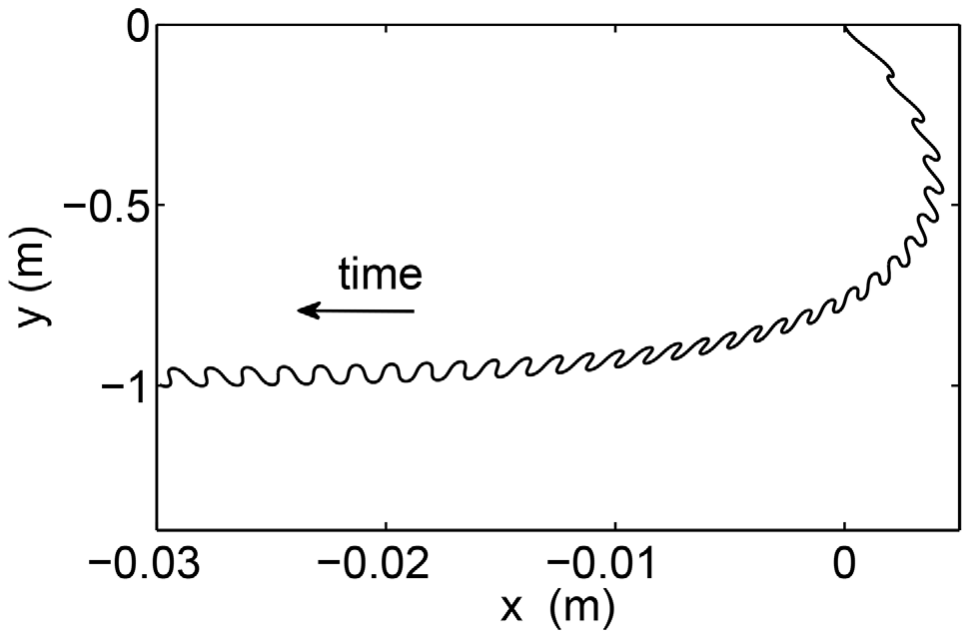
The trajectory of the center of mass of a rigid link in an external flow during passive swimming.

The external force and torque on the link could also exist because of the external flow. An external flow that has a sinusoidal transverse velocity component is considered for simplicity. No axial flow component is assumed in the surrounding fluid to eliminate the possibility of the rigid link being carried by the axial flow. The external flow provides an additional drag and moment on the link that drives its motion in the axial direction. The transverse component of the velocity of the external flow is taken as
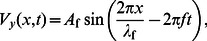
(34)in which 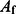 is the amplitude of the transverse velocity, 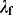 is the wavelength, and 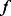 is the frequency of the traveling wave. Including the additional drag on the link due to the external flow link, the drag force Eq. (10) changes to
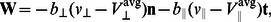
(35)in which 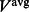 is the average of the external fluid velocity over the length of the link (in the transverse direction). It is calculated as
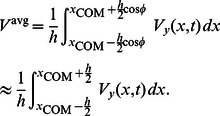
(36)

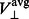
 is the component of 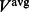 normal to the link and 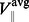 is the component of 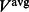 parallel to the link. The moment due to the external flow is calculated as
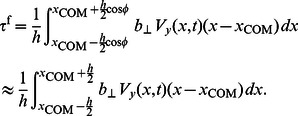
(37)Adding the additional drag and moment due to the external flow, the leading-order equations of motion for a single rigid link become
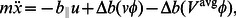
(38)

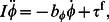
(39)

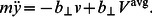
(40)Taking a time-average of Eq. (38) over a single period of swimming cycle, makes the left-hand side equal to zero and we obtain the average swimming velocity of the link at steady state as
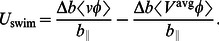
(41)To verify the equations derived for the swimming of a rigid link in the presence of an external flow, we take a rigid cylindrical rod of length 20 cm and radius 1 cm. We take the amplitude of the external flow to be 0.1 m/s and the frequency to be 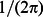 Hz. The wavelength of the fluid 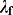 is taken to be 10 times the body length. Fig. 5 shows the trajectory of the rigid link swimming in an external flow.

#### 3.1.2 How can a fish exploit the surrounding flow field to swim passively?

The analysis done for a single rigid link in an external flow is extended to that for a flexible body in an external flow. A flexible body is kept in an external flow which does not have any mean axial velocity. It has a transverse component of velocity as given by Eq. (34). To derive the leading-order equations of motion, we calculate the drag force and the torque due to the external flow for each link and add it to Eqs. (19), (20), and (18). From Eqs. (38) and (40), it can be seen that the axial component of the drag due to the external flow for the 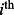 link is 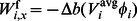 and the transverse component of drag is 
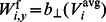
. The leading-order axial, transverse, and rotational momentum equations for the 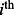 link can then be written as
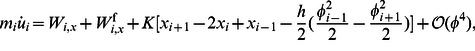
(42)

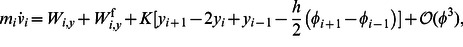
(43)

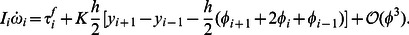
(44)The average steady swimming velocity of the flexible body in the presence of an external flow can be written as
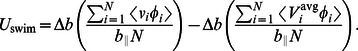
(45)


**Figure 6:**
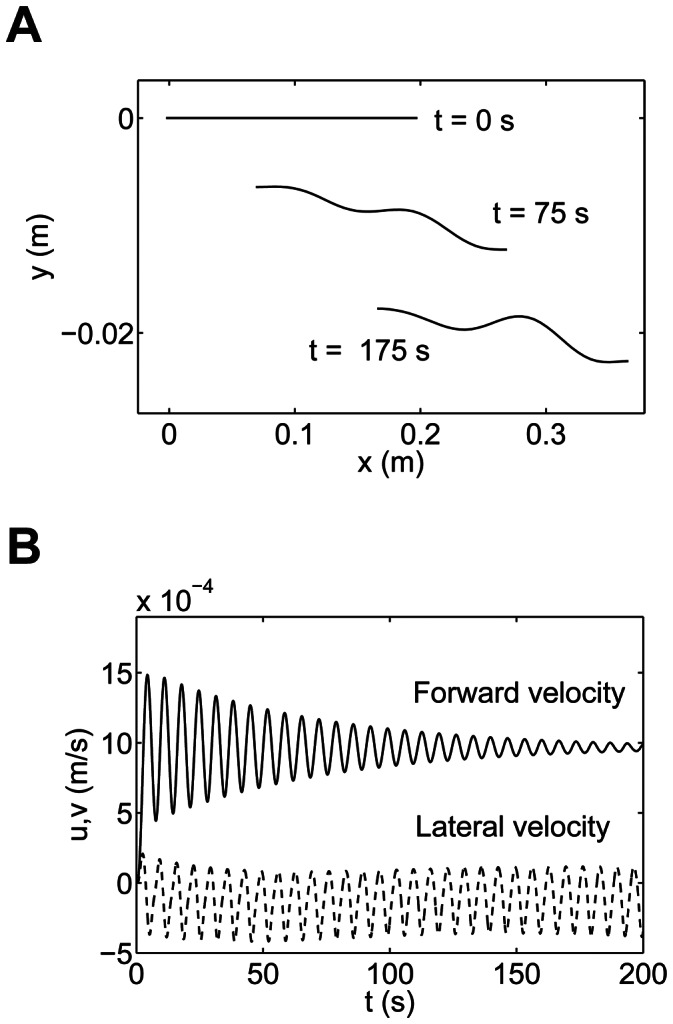
Passive swimming of a flexible body. The wavelength of the velocity change in the flow field is taken to be half the body length. (a) The position of the flexible body at different instants of time. (b) Velocity profile of the center of mass of the flexible body.

We take a uniform cross sectional body of length 20 cm and radius 1 cm. The body is discretized into 40 links of equal length. The amplitude of the velocity of the external fluid is taken to be 0.1 m/s and the frequency to be 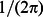 Hz. The wavelength of the fluid is taken to be half of the body length. Fig. 6(a) shows the swimming of a flexible body in an external flow. Although the external fluid flow used in this model does not mimic the vortex wake shed from the cylinders in the experiments of Liao et al. [4], it qualitatively captures the basic mechanism necessary to enable the swimming of a dead flexible animal similar to the swimming of a “dead” trout reported earlier [4], [37]. Thus, using a favorable external flow field to swim forward, a fish can decrease its muscle activity.

### 3.2 Active swimming

The term active swimming is used here to imply swimming generated by internal forcing such as muscle activation. The goal in this section is to show that active swimming is resolved within the unified framework of a forced damped oscillation formulation. To that end, we consider various aspects of muscle activated swimming. We first discuss how momentum and power is transferred during undulatory swimming. The response of the body to muscle activation in terms of its fundamental deformation modes is discussed thereafter.

#### 3.2.1 Momentum and power transfer during undulatory swimming

It can be seen from equations (18)–(20) that the lateral and rotational momentum equations provide a source term to the translational momentum. The rotational and transverse momentum equations, which can be viewed as equations that define the deformations of the body (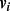 and 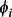), are coupled to each other, and are driven by the muscle forcing term 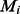. Thus, the muscle forcing creates the body deformations, which then leads to forward translation motion. This explains the momentum transfer into the translational mode.

To analyze the power transfer during steady swimming, time-averaged kinetic energy equations are derived from Eqs. (1)–(3). The time-averaged rotational kinetic energy equation is derived by multiplying Eq. (1) by 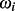, summing it over all links (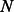 in total), and taking a time-average of the resulting equation. This is written as
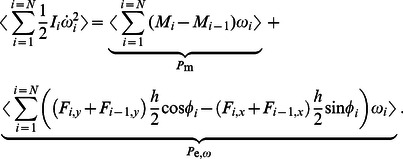
(46)Over a steady swimming cycle, the left-hand side of Eq. (46) vanishes. This implies that muscle power 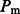 is converted to elastic power 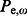 in the springs. Time-averaged transverse and axial kinetic energy equations are similarly derived:
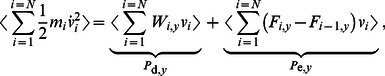
(47)

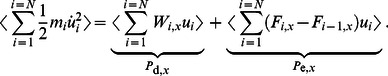
(48)The left-hand sides of Eqs. (47) and (48) vanish over a steady swimming cycle. Therefore, the elastic power in the springs in the transverse and the axial directions, 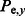 and 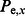, respectively (which comes from rotational kinetic energy equation through 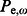 term) is dissipated by the viscous drag acting over the body in the transverse and the axial directions, 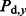 and 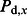, respectively. To provide a quantitative verification of the analytic expression for power transfer during free swimming, simulations were done using the nonlinear resistive chain-link PM model as described in section 2.3 (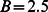 cm) for various values of the Young's modulus (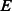). The time-averaged rotational, transverse, and axial kinetic energy equations show that the power generated by the muscles is transferred into the elastic power of the body, which is then dissipated into the fluid through the viscous drag acting on the body over a swimming cycle. Table 3 shows the mean power carried by various terms during a steady swimming cycle. Note that most of the power is input into the transverse mode. Eventually, this power is dissipated by the drag forces 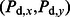. A key conclusion from this result is that, most of the power dissipation happens due to the movement in the transverse direction 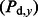 and not due to the movement in the axial direction, as is generally assumed in the efficiency measures for swimming. Thus, new efficiency metrics must take this into account while estimating the power dissipated during a swimming cycle.

**Table 3:** Table showing pathway of power transfer over a cycle of steady swimming at different values of the Young's modulus 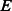.

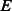 ( 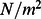 )	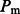 (J)	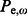 (J)	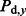 (J)	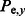 (J)	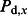 (J)	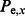 (J)
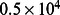	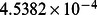	− 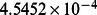	− 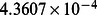	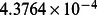	− 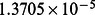	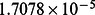
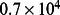	0.0011	−0.0011	−0.0010	0.0010	−7.2607 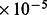	7.8884 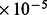
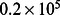	0.0094	−0.0094	−0.0063	0.0063	−0.0031	0.0031
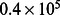	0.0163	−0.0163	−0.0100	0.0100	−0.0063	0.0063
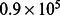	0.0090	−0.0088	−0.0071	0.0071	−0.0017	0.0017
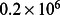	0.0057	−0.0055	−0.0051	0.0051	− 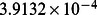	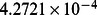

#### 3.2.2 Deformation modes of an undulatory swimmer

The transverse and the rotational equations of motion can be seen as the equations that govern the body deformations. The axial equation of motion can be seen as the equation that governs the forward translation of the body for the given deformation kinematics. Insights into how body deformations are generated can be obtained by interrogating the leading-order transverse and rotational equations of motion (Eqs. (18) and (19)). To work with the velocity variables, we differentiate these equations once more and arrange them in a matrix form as
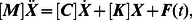
(49)in which vector 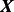


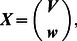
(50)is a combination of transverse velocity vector 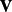 and rotational velocity vector 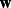, and
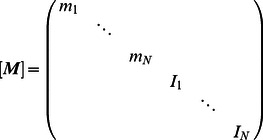
(51)is the diagonal mass matrix. In Eq. (49), 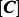 is the damping matrix, 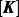 is the stiffness matrix, and 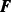
(t) is the forcing vector. The forcing vector is written as
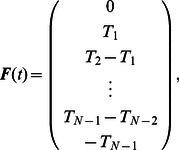
(52)in which 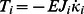 in Eq. (52). The system of second-order differential equations is converted into first-order differential equations and written as
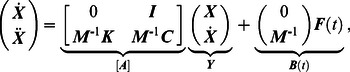
(53)which is of the form
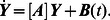
(54)Note that the first row of Eq. (53) is a trivial equation for which the left-hand side is equal to the right-hand side.

**Figure 7:**
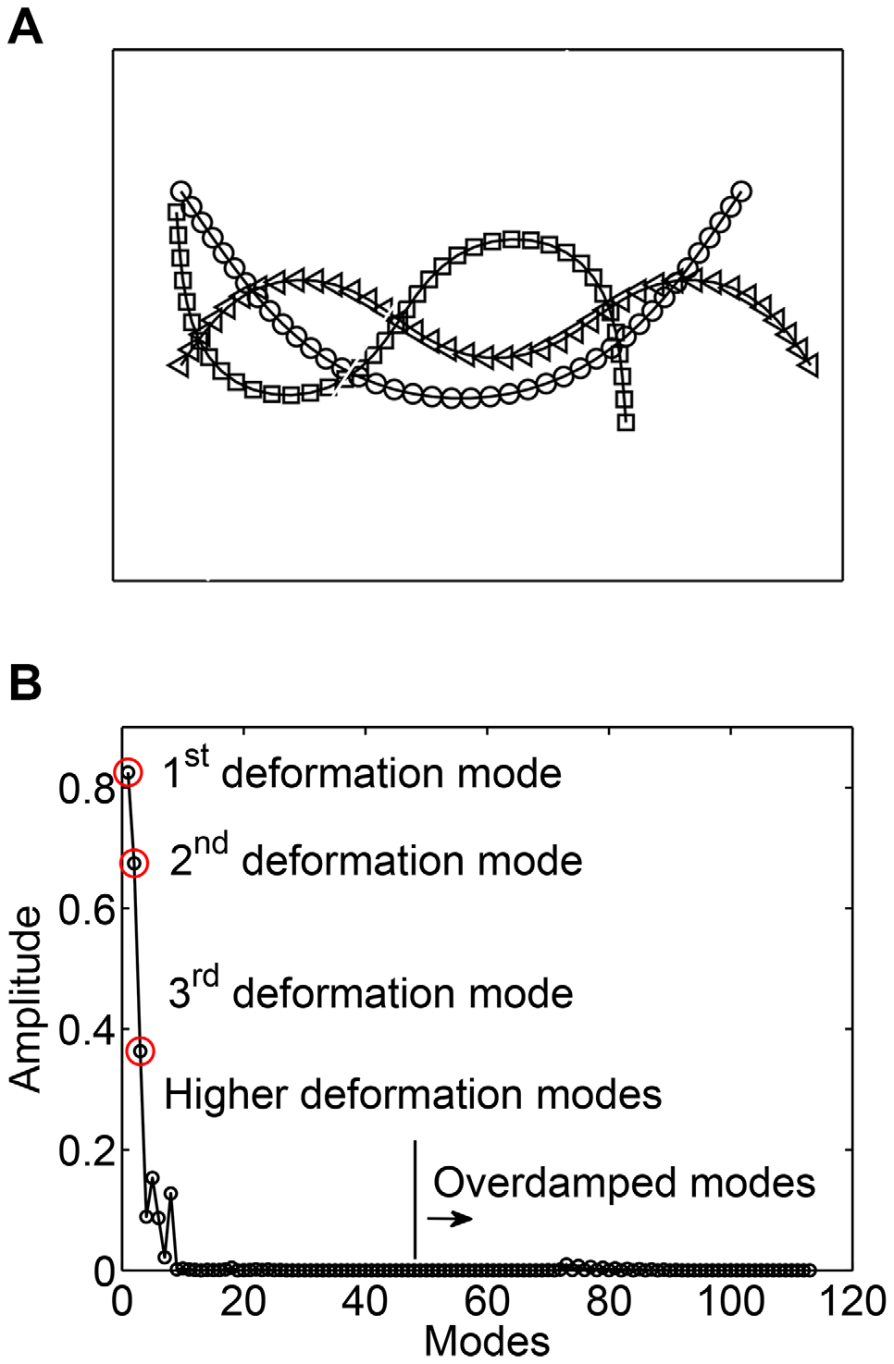
(a) The first few fundamental deformation modes of an undulatory swimmer. These modes represent the underdamped oscillatory eigenvectors of the system (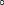: first deformation mode; 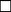: second deformation mode; 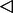: third deformation mode). (b) Amplitude or “weights” of various fundamental deformation modes during muscle activated swimming in the linear regime.

A normal mode analysis of the spring-inertia-damper system is done for unforced conditions in which the forcing term 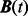 is set to zero in Eq. (54). This gives the fundamental deformation modes and the natural frequencies of this system. Shapes of the first few deformation modes are shown in Fig. 7(a). A 40-link system is considered. For the parameters considered here (section 2.5), the first 47 modes are underdamped and have a natural frequency of oscillation, i.e., the corresponding eigenvalues are complex (there are 47 complex conjugate pairs). Lower deformation modes have lower natural frequencies. Mode numbers 48 to 103 are the overdamped deformation modes, i.e., there is no natural frequency of oscillation. The elastic parameters and the stiffness coefficients are taken from [28], which were obtained based on real fish material properties. Thus, it is expected that the chosen material properties will yield realistic eigenvalues for the system. Any deformation of the body can be represented in terms of decomposition into the fundamental deformation modes of the system.

Next, a system that is forced by oscillatory muscle moments in Eq. (54) is considered. After decomposing the deformation of the body into the fundamental deformation modes, discussed above, governing equations are obtained for each of these modes. Each of the fundamental deformation modes is driven by a component of the oscillatory muscle forcing term. This is the forced damped oscillation formulation of muscle activated swimming.

The amplitude or “weight” of the emergent deformation is shown in Fig. 7(b) for each of the fundamental deformation modes. It is seen that the emergent deformation is dominantly composed of only the lower fundamental deformation modes. This is because the natural frequencies of the body corresponding to the fundamental deformation modes are higher than the muscle moment forcing frequencies. The first few deformation modes have the lowest natural frequencies (e.g. 1435.01 Hz for the first and 3889.01 Hz for the second deformation mode), whereas the physiologically relevant traveling wave frequency of muscle forcing is 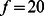 Hz [38]. This is similar to a spring-mass-damper system where the modes with natural frequencies closest to the forcing frequencies are dominantly observed.

**Figure 8:**
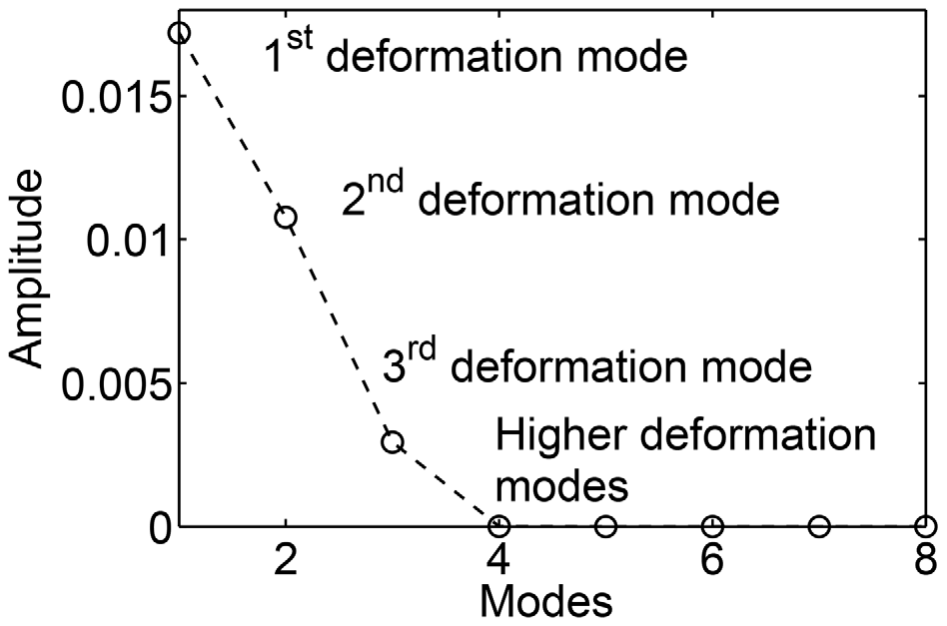
Amplitude or “weights” of Euler-Bernoulli beam deformation modes during muscle activated swimming in the nonlinear regime at Reynolds number 8000.

The conclusion above, that lower deformation modes will be typically observed, is based on a lower-order model for swimming. It helps to explain why the lower deformation modes are observed. The applicability of this conclusion is not limited to a lower-order model. In a general nonlinear problem of swimming at high Reynolds number, the eigenvalues of the system are dynamic and they change with time. This is because the coefficients of the damping matrix of the system change with time. Despite this difficulty, the conclusion above can be conceptually verified. A practically useful way to analyze such a system is to decompose the deformation of the body into the fundamental deformation modes of the Euler-Bernoulli beam equation. By superposing these modes, one can construct the shape of an undulatory swimmer at any instant as a post-processing step. To demonstrate that the conclusion above is valid even in nonlinear swimming, a case with Reynolds number of about 8000 (based on the traveling wave velocity) is simulated by using a nonlinear resistive chain-link model (same parameters of section 2.3). Fig. 8 shows the amplitude of the first few deformation modes for the undulatory swimmer at high 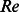. It is confirmed that only the first few deformation modes contribute most to the emergent deformation kinematics.

### 3.3 Optimal parameters for fast swimming

Parameters like muscle frequency, reference curvature, and muscle stiffness affect swimming motion. In this section we use results from the previous sections to interrogate questions related to optimal conditions that lead to fast swimming.

#### 3.3.1 What should be the frequency of the muscle activation wave?

**Figure 9:**
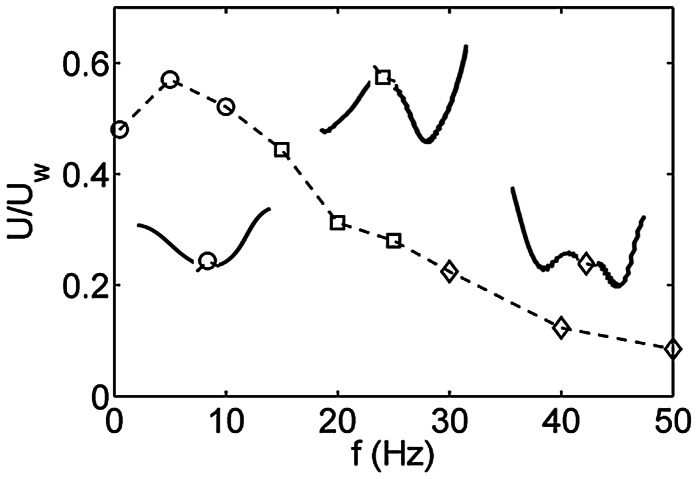
Wave efficiency (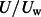) as function of the traveling wave frequency. The insets show typical body deformations at various forcing frequencies. At lower forcing frequency first few deformation modes are present. Higher deformation modes are observed at higher forcing frequencies.

In the above discussion it was argued that the value of the forcing or the traveling wave frequency 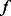 relative to the natural frequency of the damped oscillator is important for the emergent deformation characteristics of the body. Thus, it implies that as the forcing frequency is increased higher deformation modes should also be observed. To verify this, Fig. 9 shows a plot of wave efficiency (swimming velocity normalized by the wave velocity 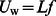; in which 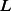 is the body length) as a function of the forcing frequency. All cases were solved by using the nonlinear resistive chain-link PM model. All physical parameters were taken from section 2.3 except for the forcing frequency which was varied. It is seen that at higher frequencies, higher deformation modes are triggered which is consistent with the discussion above (see insets of Fig. 9). Higher deformation modes do not represent the best kinematics to push the fluid back and thus propel the body forward. Hence, the normalized swimming velocities are increasingly lower as the forcing frequency is increased.

#### 3.3.2 What are the optimal deformation kinematics for fast swimming?

According to Eq. (24) an undulatory swimmer that maximizes 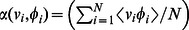 will swim the fastest. Since 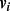 and 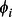 define the deformation kinematics of the body, 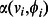 primarily depends on the deformation of the swimming body. This expression, although derived in the leading-order regime, captures the effect of the local lateral velocity 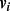 and the local inclination 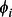 of the body segments on the axial swimming velocity. The applicability of this expression in the nonlinear regime is tested by performing fully resolved simulations on a three-dimensional eel model. An adaptive immersed body framework is used to perform these simulations [30]–[32], [39].

We use an approach similar to that of Kern and Koumoutsakos [24], who search for the best deformation kinematics within a family of deformations. The deformation kinematics are defined by five control parameters that give the curvature of the backbone of the eel. The curvature is defined along the arclength 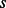 of the backbone of length 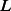 as
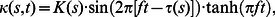
(55)in which 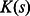 is a cubic polynomial with values of 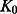, 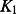, 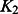, 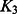 at 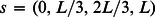, 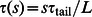, and 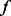 is the frequency of the traveling wave. With the choice of five parameters 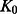, 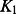, 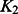, 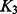, and 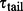, a wide range of motion patterns can be generated [24]. Using the curvature information a unit-speed curve is obtained. The objective function 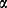 is maximized for the unit-speed curves by varying the five control parameters by solving a constrained optimization problem using the fmincon function in MATLAB. Note that no solution of the swimming motion is required unlike the original optimization by Kern and Koumoutsakos [24]. The initial guess or the reference values for the control parameters are taken to be the same as those from [24], which are 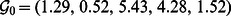. The value of 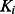 is bound between 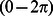 and the value of 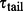 is bound between 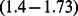, as was done in [24]. The optimized values of the control parameters as reported in [24] are 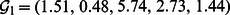. The values of 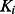 and 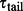 corresponding to the maximum value of 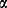 are 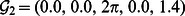.

The value of 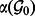 is 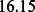, 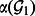 is 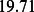, and 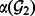 is 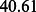. Thus, 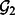 should give the fastest swimming velocity; even faster than the kinematics identified by [24] based on fully resolved simulations. To confirm this, fully resolved simulations were performed by taking the kinematics obtained from 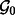, 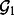, and 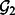. The physical parameters used were identical to those in [24]. An efficient constraint based immersed body method [39]–[41] implemented in the IBAMR software framework [42] is used. The combined momentum equation for the fluid and immersed structure is solved on a block-structured Cartesian grid, where higher grid resolution is deployed only near the immersed structure and the vortex structures shed from such interfaces. Four grid levels were used in the domain, with the coarsest level, 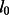, having 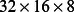 grid cells. A refinement ratio of 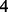 was used for levels 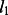 and 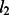, and a ratio of 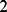 for level 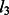. Regions of space where the vorticity magnitude exceeded 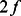 were additionally tagged. The domain size was taken to be 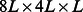, in which 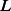 is the body length. Adaptive time stepping dictated by convective Courant-Friedrichs-Lewy (CFL) number of 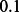 was used. Numerical integration of Eq. (55) to obtain unit-speed curve was done using GSL [43] library.

**Figure 10:**
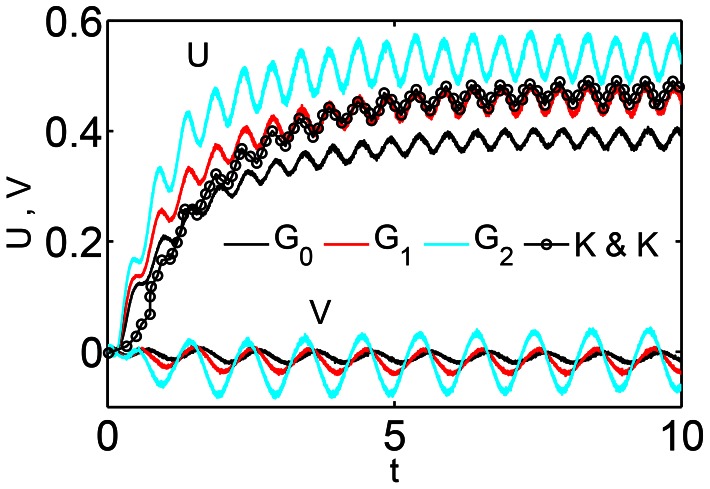
Fully resolved simulations of three-dimensional eel for the kinematics cases 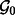, 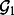, and 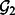. The figure shows normalized axial 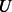 and lateral 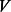 velocities (normalized by the wave speed, 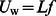) of the center of mass of the eel as a function of normalized time 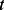 (normalized by 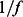). Upper: axial velocity; Lower: transverse velocity. In the figure 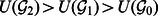. (–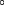
–) 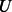 profile for 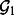 from Kern and Koumoutsakos (K & K) [24].

**Figure 11:**
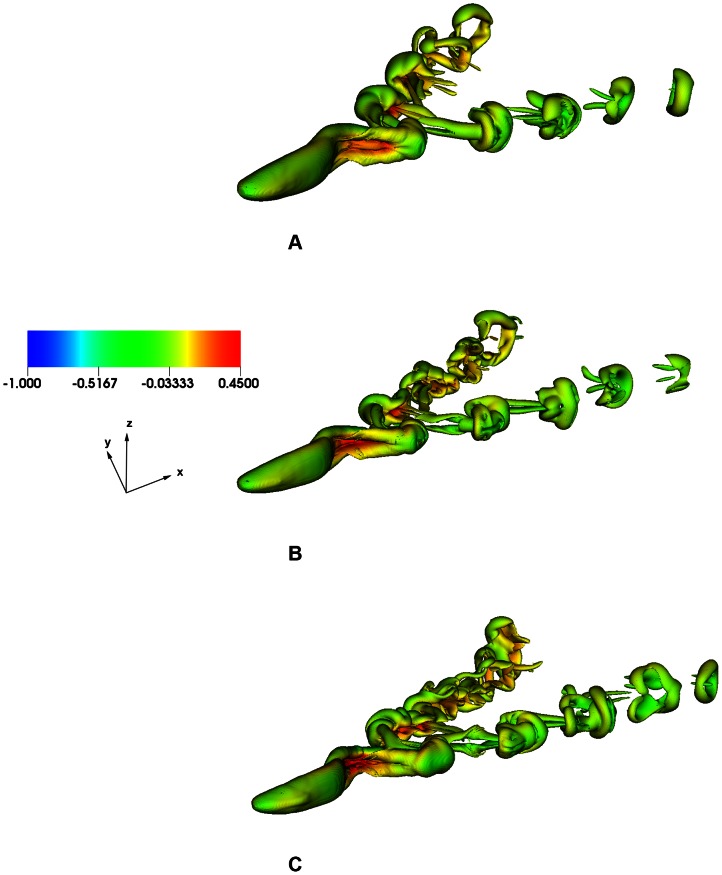
Comparison of three-dimensional flow structures for an eel swimming with kinematics given by (a) 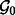, (b) 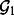, and (c) 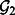 at time 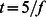. Isosurface of normalized vorticity (normalized by 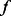) magnitude of 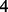 and color coded by normalized transverse velocity (normalized by 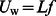) is shown.

Fig. 10 shows the axial and lateral velocities (normalized by the wave speed, 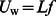) of center of mass of the eel as a function of time (normalized by 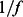). It is seen that the proposition that the kinematics that maximized 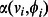 would also maximize the speed of the swimmer is valid even for nonlinear high Reynolds number problems. Fig. 11 compares the isovorticity contour for an eel swimming according to the kinematics given by 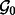, 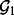, and 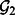 at time 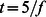. As can be seen in the figure, the stronger vortex wake for 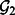 kinematics results in a faster swimming speed of the eel as compared to 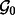 and 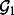 kinematics. The observed flow pattern is also consistent with prior results [24] (see Videos S1 and S2 in Supporting Information for the time evolution of flow features).

#### 3.3.3 How stiff should muscles become for optimal swimming?

According to Eq. (6), muscle activated moment is proportional to the Young's modulus 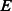 of the body. A stiffer body has a higher value of the Young's modulus, which helps the body to match its preferred curvature 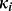. Thus, the swimming speed depends on 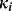 and the bending stiffness of the body represented by 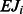 (Eq. (6)).

If the body is very stiff then the observed kinematics closely follow 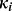. This will be shown below. In this case, optimality for fast swimming is achieved if 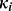 corresponds to the optimal kinematics found, for example, in the previous section for fast swimming.

Tytell et al. [1] reported optimal body stiffness that led to maximum swimming velocity. Their results were based on simulations that used fully resolved fluid-elastic body interactions. Here, those results are qualitatively reproduced and explained using the reduced-order model. All cases were solved using the nonlinear resistive chain-link model. All physical parameters were the same as those in section 2.3 except for the Young's modulus 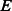, which was varied. Fig. 11 shows a plot of the swimming velocity as a function of 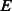 (or body stiffness 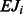). As reported by Tytell et al. [1], there is an optimal stiffness at which the swimming velocity is maximized. It is shown below that the preferred curvature of the body 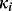 that results from muscle activation is important to explain this trend.

**Figure 12:**
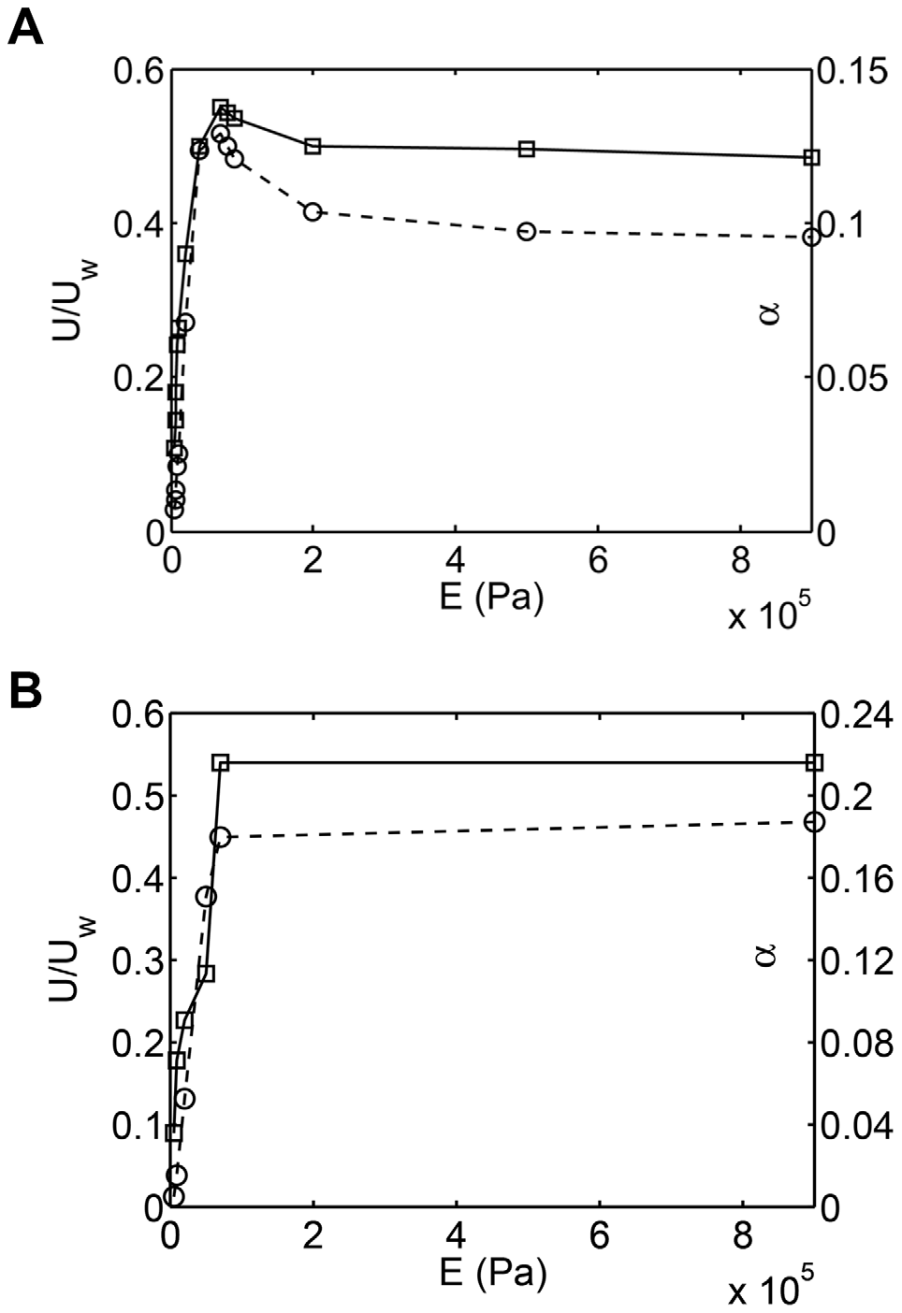
(a) Normalized swimming speed (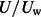) (— 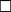
—, left vertical axis) and the objective function 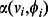 (–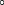
–, right vertical axis) as a function of Young's modulus 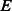 (or equivalently bending stiffness 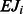 for constant 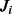). The preferred curvature 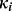 of a traveling wave is used to actuate the swimming motion. (b) Normalized swimming speed (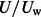) (—
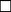
—, left vertical axis) and the objective function 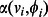 (–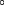
–, right vertical axis) as a function of Young's modulus 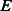 (or equivalently bending stiffness 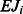 for constant 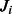). The curvature at the optimal condition of (a) is used as the preferred curvature 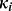 to actuate the swimming motion.

At low stiffness, the natural frequencies of the body are lower. Hence, a given forcing frequency triggers higher deformation modes of a less stiff body (see insets Fig. 9). In other words, the body appears floppy and is not able to efficiently propel itself forward due to the presence of higher deformation modes. At high stiffness, the observed deformation kinematics are nearly the same of those due to 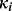. If the imposed 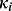 does not represent the best deformation kinematics for fast swimming, then at high stiffness the swimming velocity is lower than optimal. At intermediate stiffness the body kinematics are optimal for fast swimming, leading to the trend shown in Fig. 12(a). It is also noted that the swimming velocity is practically unchanged with any further increase in body stiffness beyond a certain value (Fig. 12(a)). This can be explained by noting that the body kinematics remain the same as those imposed by 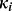 beyond a certain body stiffness. Fig. 12(a) also shows a plot of 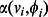 as function of 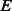. It is seen that the trend in 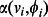 is consistent with the trend of the swimming velocity. As seen earlier 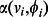 directly correlates with the deformation kinematics. Thus, the observed trend in swimming velocity in Fig. 12(a) is a consequence of the deformation kinematics that the body acquires as a function of its stiffness.

To further verify the importance of 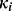, the deformation kinematics at the optimal condition in Fig. 12(a) were noted. Then another set of simulations were done with 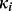 equal to these optimal kinematics. In this case, since 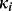 now represents optimal kinematics, once the body becomes stiff and follows 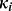, the swimming velocity should not reduce. Thus, the swimming velocity trend should plateau as a function of 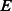 instead of showing an optimal (i.e. a maximum) point. This is confirmed in Fig. 12(b). Once again, the trend in 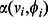 is found to be consistent with the trend in swimming velocity.

## Discussion

In this work our primary thesis is that the deformation is a result of some forcing which can be caused by muscles or the surrounding fluid or both. We have used a forced damped oscillation framework to formulate the problem of swimming. This is in the same spirit as the damped harmonic oscillator framework that has been applied in the past to analyze numerous engineering systems such as the tuned mass damper used in power transmission, automobiles, and buildings to reduce vibrations.

The results presented above validate several hypotheses that show the utility of the forced damped oscillation framework. These examples (points 1–4, below), which provide new insights, are discussed below.

1. Our hypothesis was that the forced damped oscillation framework would provide unified insights into how the body deformation emerges and subsequently how the body deformation leads to swimming movement. First, the problem formulation and the results discussed earlier validate this hypothesis where it is seen that body deformations emerge as a superposition of various deformation modes in response to some forcing. This is similar in principle to how various deformation modes emerge on a guitar string or a fluttering flag. Second, insights into how body deformations lead to forward swimming movement is represented by Eq. (24). As hypothesized, this equation provides a unified insight into how forward swimming momentum is obtained, no matter how the system is forced. Thus, active and passive swimming scenarios are understood in a unified way. It is to be noted that although Eq. (24) was based on a simplified model, the results were verified from fully resolved computational fluid dynamic simulations of optimal gaits for fast swimming.

2. Our hypothesis was that the forced damped oscillation framework will provide insights into why relatively simple forcing patterns can trigger seemingly complex deformation kinematics that lead to movement. This was validated from our results where we show how forcing triggers different deformation modes of the body. The presence of multiple deformation modes makes the body deformation appear complex.

3. We hypothesized that the proposed framework will explain why lower deformation modes (i.e., fewer number of waves on the body) are typically observed in undulatory swimming in nature. This was resolved by our results where we show how the value of the forcing frequency relative to the natural frequencies of the body is important to trigger the various deformation modes of the body (see Fig. 9). Typical forcing frequencies in nature are lower than the typical resonance frequencies of the damped oscillator (i.e., the fish body). Hence, it is not surprising that lower deformation modes are observed in nature. In anguilliform swimmers, the body is long and slender. As a result the first few deformation modes would be triggered in these swimmers. However, carangiform, sub-carangifrom, and thunniform swimmers have relatively short and stiff bodies. In such swimmers, the body deformations are predominant in the caudal fin region indicating lower deformation modes compared to anguilliform swimmers, as expected from our results.

4. The proposed framework helped identify parameters for which swimming is viable. The specific parameters that were identified include appropriate frequencies for swimming (Fig. 9), appropriate body stiffness for swimming (Figs. 12(a) and 12(b)), and appropriate gaits for fast swimming (Figs. 10 and 11). The forced damped oscillation framework was crucial to obtain key insights into each of these parameters. For example, the forcing frequencies at which swimming would be viable are those that do not trigger the higher deformation modes (Fig. 9). Thus, high frequencies relative to the natural frequencies are not appropriate for swimming. Similarly, for a given forcing frequency the body should be stiff enough for swimming to be possible (Figs. 12(a) and 12(b)). This is because higher stiffness leads to higher natural frequencies compared to the forcing frequency which in turn would not trigger the higher deformation modes that are not desirable for swimming. Finally, the gaits that maximize the forward momentum transfer according to Eq. (24) are most appropriate for fast swimming. It was found that higher deformation modes do not work well according to this metric. These results were confirmed from fully resolved simulations (Fig. 10).

The results in point 4, above, are practically the most important since they have biological as well as engineering relevance. The practical utility of the above results in engineering is apparent if one considers the design of a biomimetic underwater robotic vehicle. The above analysis helps identify material properties as well as viable frequencies of an undulator that would drive the vehicle.

The above results may also provide useful insights in evolutionary biology. In particular consider the issues pertaining to the evolutionary emergence of certain species. To that end consider the evolution of aquatic animals that use the undulatory mode for propulsion. Assume that only those undulatory animals that can swim effectively would have survived. If so, then it is of interest to know if specific parameters such as body stiffness and activation frequency of the muscles are crucial for survival. Based on the results above, one could hypothesize that only those species with appropriate muscle activation frequency (low; e.g., Fig. 9) and body stiffness (high; e.g., Fig. 12(b)) would survive and evolve. Thus, this analysis helps identify the key mechanistic parameters that would have been crucial to the evolutionary emergence of undulatory swimmers. While our study certainly does not directly consider evolution, it points to the ways to combine analyses such as ours with evolutionary biology to explore what impact the mechanics of movement may have played on the emergence of certain animals.

Similarly, these results could be potentially used to gain insights into the ability to adapt through evolution. As an example, consider a hypothetical scenario where the ability to move is dependent on having a very specific deformation of the body which in turn is very sensitive to the muscle activation pattern. In such a scenario, any change in the parameters would seriously hamper the ability of that animal to move. Such an animal would not be very robust in its ability to move in a changing environment. In addition, it is very likely that any small changes to physical characteristics from one generation to next (and through evolution) would have a detrimental effect on the survival of that species. Our results suggest that in reality the above scenario may not occur. We find that the ability to swim, while dependent on the mechanistic parameters, may not be very sensitive to it. For example, results in Fig. 12(b) indicate that as long as the body stiffness is above a certain value, the ability to swim fast is insensitive to the value of stiffness. Similarly, as long as the frequency is below certain value (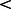
30 Hz for the parameters used in Fig. 9), the wave efficiency is not significantly affected, once again suggesting some degree of robustness to changes in the parameters. Finally, the result that simple forcing patterns can lead to swimming movement is also suggestive of robustness in the sense that it is not essential to have a very specific and complex forcing pattern to make swimming viable. One may therefore hypothesize that the relative insensitivity to parameters, and the noncomplexity of the feasible forcing patterns would be beneficial for adaptation through evolution. Although we do not directly interrogate this issue of adaptation, such hypotheses motivated by the mechanics of movement warrant further investigation.

The proposed framework leads to an understanding of many key parameters that are critical to movement. In turn, it has the potential to provide further insights into how movement can be controlled in biomimetic underwater vehicles and analogously to decipher how neuronal control of movement might function in animals.

In this work we also quantified the pathway of power transfer from muscles to forward swimming. It is shown that over a steady swimming cycle, the net power generated by the muscles is transferred into the elastic power of the body which is then dissipated into the fluid while undulating the body (Table 3). Identifying this pathway is crucial to obtain a measure for useful work during swimming. This in turn can be used to estimate useful measures for swimming efficiency. This result is important because the efficiency of undulatory swimmers is conventionally defined as the ratio of power spent in overcoming hydrodynamic drag in the direction of swimming to the total muscle power produced. “Gray's paradox” [44]–[46] was based on this definition of efficiency where it was paradoxically found that the drag power was greater than the muscle power. Although the paradox is now considered resolved, our results suggest that the assumption, underlying Gray's result, that the useful power is the one spent to overcome drag in the swimming direction may not be appropriate. The useful power is in fact the one spent to undulate the body. Thus, “Gray's paradox” should be revisited in the context of our results on the pathway of power transfer.

In summary, we applied the forced damped oscillation framework to undulatory swimming to understand the emergence of movement due to muscular and/or environmental forcing.

## Supporting Information

Video S1Video S1 shows a three-dimensional vortex wake behind the eel, swimming with 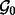 kinematics, as given in the section 3.3.2(MPEG)

Video S2Video S2 shows a three-dimensional vortex wake behind the eel, swimming with 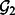 kinematics, as given in the section 3.3.2. The 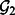 kinematics produces a stronger wake structure as compared to the 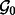 kinematics. Hence, it results in a faster swimming velocity for the eel. Also notice the typical V-pattern wake behind the eel. It results from the vortex-shedding at tip of the tail during each half-cycle of swimming.(MPEG)
